# A Noncanonical Auxin-Sensing Mechanism Uncovered by Screening the Auxin Response Factor 3 Interacting Proteins in Tomato

**DOI:** 10.3390/ijms27031227

**Published:** 2026-01-26

**Authors:** Lin Wang, Xirong Yang, Sidratul Muntha, Liepeng Dong, Qingmin Xie, Taotao Wang, Chunmei Shi, Changxian Yang

**Affiliations:** 1 National Key Laboratory for Germplasm Innovation and Utilization of Horticultural Crops, Huazhong Agriculture University, No. 1 Shizishan Street, Hongshan District, Wuhan 430070, China; wang_lin@webmail.hzau.edu.cn (L.W.);; 2 Peking University Institute of Advanced Agricultural Sciences, Shandong Laboratory of Advanced Agricultural Sciences in Weifang, No. 699 Binhu Road, Xiashan Eco-Economic Development Zone, Weifang 261325, China; 3 Energy-Rich Compound Production by Photosynthetic Carbon Fixation Research Center, Shandong Key Lab of Applied Mycology, College of Life Sciences, Qingdao Agricultural University, No. 700 Changchen Road, Qingdao 266109, China; 4 College of Horticulture and Landscape Architecture, Yangzhou University, Yangzhou 225009, China; 008392@yzu.edu.cn

**Keywords:** auxin response factor 3, yeast two-hybrid screen, interacting proteins, parthenocarpy, tomato

## Abstract

Within the canonical auxin signaling pathway, Auxin Response Factors (ARFs) are transcriptionally repressed by AUX/IAA proteins under low auxin conditions, and this repression is alleviated as auxin concentrations increase. By contrast, ARF3 functions as a central regulator of gynoecium morphogenesis in Arabidopsis via a non-canonical auxin-sensing mechanism that relies on dose-dependent modulation of its protein–protein interaction network. To investigate whether an analogous regulatory mechanism operates in tomato (*Solanum lycopersicum*), we identified the tomato ARF3 homolog (SlARF3) and utilized it as bait in a yeast two-hybrid (Y2H) screen. This screening approach yielded 137 positive clones, corresponding to 118 putative interacting proteins. Notably, all of these interactions were abolished in the presence of 3-indoleacetic acid (IAA), indicating that SlARF3 engages in auxin-sensitive protein–protein interactions and thereby mediates auxin-dependent signal transduction. Among these, we identified an auxin-sensitive interaction between SlARF3 and TM29, a central regulator of parthenocarpy, underscoring its critical role in this developmental pathway. Functional analyses further demonstrated that silencing *SlARF3* induces parthenocarpic fruit formation. Taken together, these findings define a previously uncharacterized SlARF3-centered interaction network and provide a conceptual framework for elucidating non-canonical auxin signaling pathways underlying tomato development.

## 1. Introduction

Auxin Response Factors (ARFs) are key transcriptional regulators in the auxin signaling pathway [1,2,3]. They act as sequence-specific DNA-binding proteins that recognize TGTCNN-type Auxin Response Elements (AuxREs) within the promoters of target genes, thereby directly controlling the transcription of primary auxin-responsive genes [4,5]. The activity of ARFs is suppressed by AUX/IAA repressor proteins. In the presence of auxin, AUX/IAAs associate with the SCF^TIR1/AFB^ E3 ubiquitin ligase complex, which promotes their polyubiquitination and subsequent degradation by the 26S proteasome. The removal of AUX/IAAs lifts repression on ARFs, activating the auxin-dependent transcriptional program [6,7,8,9].

Most ARFs exhibit a conserved domain structure, comprising an N-terminal DNA-binding domain (DBD), a middle region (MR) that harbors either an activation domain (AD) or a repression domain (RD), and a C-terminal Phox and Bem1 (PB1) domain responsible for mediating protein–protein interactions [10,11,12]. In Arabidopsis, ARF3, ARF13, ARF17, and ARF23 do not possess the PB1 domain, which prevents their association with AUX/IAA proteins in the canonical auxin signaling pathway [13]. Instead, AtARF3 carries a C-terminal ETT-specific (ES) domain that can directly perceive auxin. Auxin binding to this domain triggers the dissociation of AtARF3 from its interaction partners, thereby modulating the expression of AtARF3 target genes [14].

In Arabidopsis, mutations in *ARF3* interfere with several facets of reproductive development. The observed defects span core processes, including meristem maintenance and floral determinacy, as well as the formation of particular organs, such as gynoecium morphogenesis, ovule development, and self-incompatibility [15,16,17,18]. In tomato, 22 *ARF* genes have been identified so far [19]. SlARF3 (Solyc02g077560) is phylogenetically grouped with its *Arabidopsis thaliana* ortholog, AtARF3, indicating that they most likely retain similar structural and functional properties [20]. SlARF3 has been shown to be crucial for trichome formation and leaf morphogenesis [20,21]. Nonetheless, the role of SlARF3 in tomato pistil development is still unknown, and studies investigating its protein interaction network remain limited.

The absence of a canonical PB1 domain in SlARF3 necessitates the systematic identification of its protein interactors to define the mechanistic basis of its unique, non-canonical auxin signaling. Thus, a comprehensive understanding of SlARF3 function relies heavily on characterizing its protein interaction partners. With the continual advancement of Y2H methodologies, numerous studies have used Y2H library screening to identify putative interactors of key plant proteins [22,23,24]. Using this strategy in tomato, we carried out an extensive Y2H screen and identified 118 candidate proteins that may interact with SlARF3. Notably, auxin disrupted all of these interactions, strongly implying that SlARF3 directly senses auxin signals in tomato via protein–protein interactions. In particular, we discovered that SlARF3 associates with TM29 to modulate parthenocarpy in tomato. Collectively, these interacting proteins offer valuable leads for further elucidating the biological roles of SlARF3 in tomato.

## 2. Results

### 2.1. Identification of the SlARF3 in Tomato

**Figure 1:**
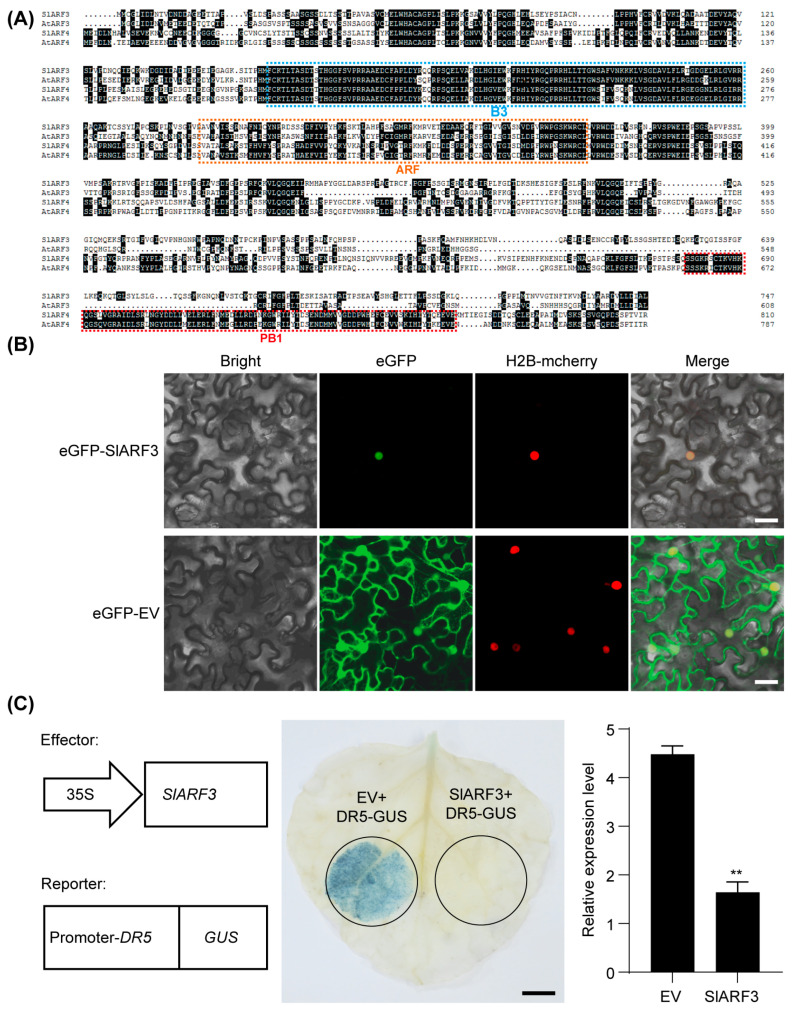
Functional characterization of SlARF3. (**A**) Amino acid sequence polymorphism analysis of ARF3 and ARF4 in tomato and Arabidopsis. Domain structures are schematically represented by dashed boxes: B3 domain (blue), ARF domain (orange), and PB1 domain (red). Black background indicates the conserved residues. (**B**) Subcellular localization analysis of SlARF3. eGFP-SlARF3 and H2B-mcherry were co-expressed in *Nicotiana benthamiana* leaves and imaged in their respective fluorescence channels by confocal laser scanning microscopy at 48 h post-agroinfiltration. eGFP-EV was the negative control. Bars, 25 μm. (**C**) The effect of SlARF3 on auxin signaling inhibition. Transcriptional activation assay by co-expression of the effector (35S: *SlARF3*) and reporter (*ProDR5: GUS*) in *Nicotiana benthamiana* leaves, followed by GUS staining and qRT-PCR of leaves collected 60 h post-infiltration. The relative expression level corresponds to the *GUS* expression driven by the *DR5* promoter. Data are presented as means ± SD (n = 3), **, *p* < 0.01 (Student’s *t*-test). Bar, 1 cm.

A thorough understanding of SlARF3’s functional characteristics is essential for deciphering its protein–protein interaction network. Therefore, we initiated our investigation with a systematic functional analysis of this protein. Phylogenetic analysis clustered SlARF3, AtARF3, SlARF4, and AtARF4 into the same clade, indicating their closest phylogenetic relationship [20]. However, sequence alignment revealed that while SlARF4 and AtARF4 contain the canonical B3, ARF, and PB1 domains, both SlARF3 and AtARF3 lack the PB1 domain, suggesting that they may possess atypical ARF functions (Figure 1A). To determine the subcellular localization of SlARF3, we fused eGFP to its N-terminus. Fluorescence imaging showed that the fusion protein was confined exclusively to the nucleus together with the nuclear localization marker (Figure 1B). To further probe its primary role in the auxin signaling pathway, *SlARF3* under the control of the 35S promoter was transiently co-expressed with the auxin-responsive β-glucuronidase (GUS) reporter *ProDR5: GUS* in *Nicotiana benthamiana* leaves. Strikingly, co-expression of *SlARF3* markedly suppressed *DR5*-driven GUS activity (Figure 1C), demonstrating that SlARF3 functions as a negative regulator in auxin signaling. To characterize the spatial expression pattern of *SlARF3*, we measured its transcript abundance in roots, stems, leaves, flowers, and fruits. *SlARF3* transcripts were detected in all tested tissues, with particularly high levels in leaves and flowers, pointing to an important role in these organs (Figure S1).

### 2.2. Y2H Library Construction

Considering that SlARF3 may fulfill multiple roles in diverse tomato tissues, a Y2H cDNA library was generated from a composite pool of root, stem, leaf, flower, and fruit tissues. mRNA from these tissues was combined and used as a template for first-strand cDNA synthesis. The product of cDNA amplification was subsequently ligated into the pGADT7 vector and introduced into *Escherichia coli* TOP10 competent cells by electroporation to construct the Y2H library. The library titer was estimated to be approximately 2.0 × 10^8^ colony-forming units per milliliter (CFU/mL) (Figure S2A). PCR amplification of inserts from twenty-four randomly selected transformants revealed an average insert size of approximately 1000 to 2000 bp (Figure S2B).

### 2.3. Screening of SlARF3 Interactors

**Figure 2:**
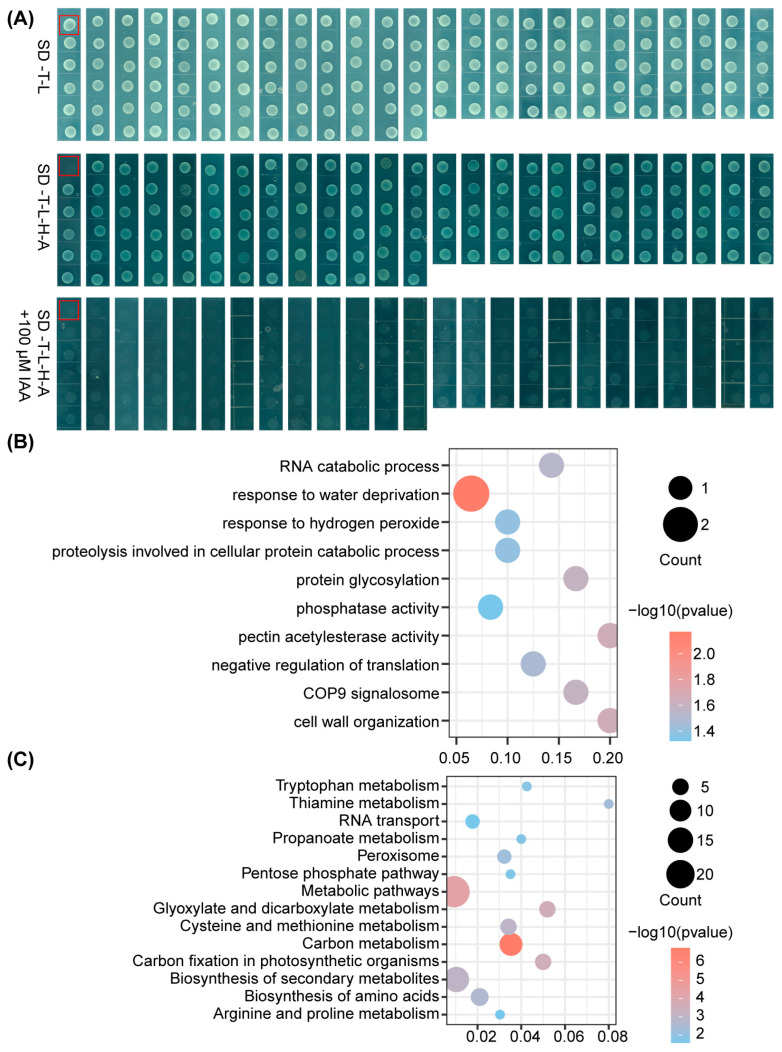
Identification of SlARF3-interacting proteins via Y2H screening and GO/KEGG enrichment analyses of candidate interactors. (**A**) Y2H screening for SlARF3-interacting proteins. Positive yeast transformants co-transformed with the bait plasmid BK-SlARF3 and the library plasmid were re-grown on both SD-T-L and SD-T-L-H-A selection media. Parallel sets of SD-T-L-H-A plates were supplemented with 100 μM IAA to assess the potential influence of auxin on these protein–protein interactions. Red boxes indicate AH109 co-transformed with BK-SlARF3 and the empty pGADT7 vector, serving as the negative control. (**B**) GO enrichment of SlARF3-interacting proteins. (**C**) KEGG pathway enrichment of candidate interactors. In GO and KEGG enrichment plots, the horizontal axis represents the gene ratio. The size of each node is scaled to the number of input genes assigned to that term, and its color corresponds to the −log10 (*p*-value).

The BK-SlARF3 bait construct was first generated and subsequently co-transformed with pGADT7 into the *Saccharomyces cerevisiae* strain AH109. The resulting transformants were able to grow on synthetic dropout (SD) medium lacking tryptophan and leucine (SD-T-L), but failed to grow on SD medium lacking tryptophan, leucine, histidine, and adenine (SD-T-L-H-A) (Figure S3A). These findings demonstrate that SlARF3 does not possess intrinsic transcriptional activation activity in the AH109 yeast assay system. Subsequently, the AH109 yeast strain transformed with BK-SlARF3 was employed for Y2H screening. A tomato Y2H cDNA library was introduced into competent yeast cells containing BK-SlARF3, and the transformants were selected on SD-T-L-H-A (Figure S4A). In total, 137 putative positive colonies were recovered. These positive clones were lysed, subjected to PCR amplification using specific primers (Figure S4B), and subsequently sequenced with T7 primer. The screening analysis yielded a total of 118 distinct candidate proteins that are predicted to interact with SlARF3 (Table S1). Comparable growth was observed on SD-T-L medium regardless of IAA, demonstrating its absence of toxicity in this yeast system (Figure S3B). Notably, all these interactions were abolished upon supplementation of the SD-T-L-H-A medium with IAA, indicating that the identified proteins interact with SlARF3 in an auxin-sensitive manner (Figure 2A).

To obtain a functional characterization of the candidate interactors identified in the Y2H screen, we conducted Gene Ontology (GO) and the Kyoto Encyclopedia of Genes and Genomes (KEGG) enrichment analysis. GO analysis revealed enrichment in biological processes related to stress adaptation and protein catabolism, along with cellular component terms encompassing the COP9 signalosome, implicating potential functions in protein complex regulation and turnover (Figure 2B). In parallel, KEGG analysis highlighted that the interactors were significantly enriched in core metabolic pathways, particularly those associated with amino acid biosynthesis, carbon metabolism, and secondary metabolite production, suggesting their role as critical nodes integrating primary and specialized metabolism (Figure 2C). Taken together, these findings suggest that SlARF3-interacting proteins are functionally linked to both metabolic regulation and adaptive signaling pathways and may act as molecular integrators that translate developmental and environmental cues into specific cellular responses.

### 2.4. Subcellular Localization of the Interactors of SlARF3

**Figure 3:**
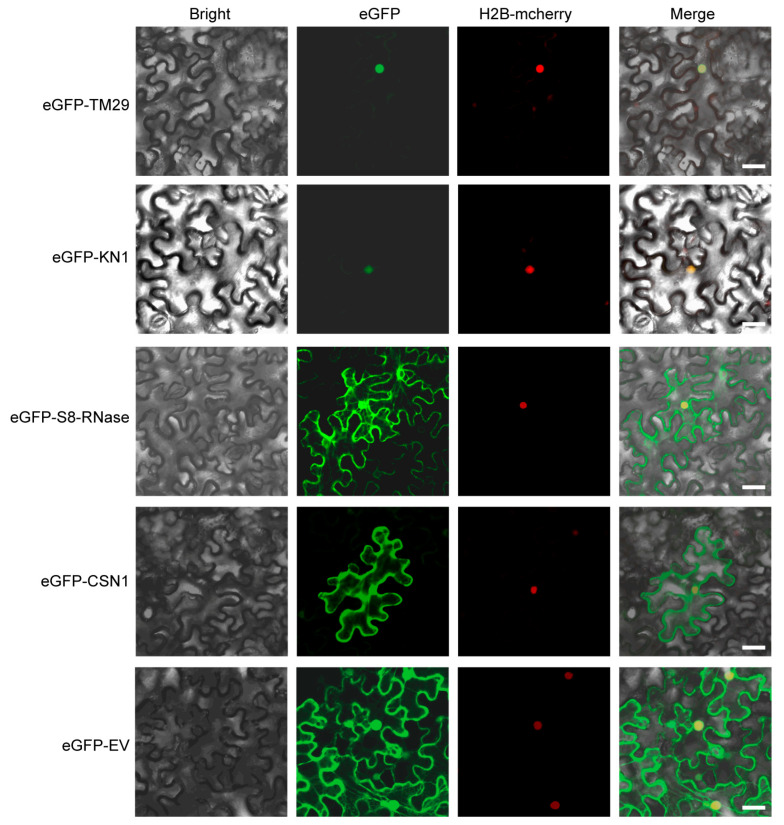
Subcellular localization analysis of SlARF3 interacting proteins. eGFP-interactors and H2B-mcherry were co-expressed in *Nicotiana benthamiana* leaves via agroinfiltration. Images of epidermal cells were captured at 60 h post-infection using a confocal laser scanning microscope. eGFP-EV was used as a negative control. H2B-mcherry was used as a nuclear marker. Bars, 25 μm.

To rigorously assess the interaction patterns between SlARF3 and its putative protein partners, four candidate interacting proteins that were putative auxin-related were selected for detailed analysis. To investigate the subcellular localization of TM29, KN1, S8-RNase and CSN1, the corresponding full-length coding sequences (CDSs) were amplified, cloned, and fused in-frame to the N-terminus of enhanced green fluorescent protein (eGFP). *Agrobacterium tumefaciens* strains harboring constructs for the expression of *eGFP*, *eGFP-TM29*, *eGFP-S8-RNase*, *eGFP-CSN1*, and *eGFP-KN1* were subsequently used for transient expression in *Nicotiana benthamiana* leaves via agroinfiltration. As shown in Figure 3, all fusion proteins showed a nuclear distribution pattern, analogous to that of SlARF3 (Figure 1B), indicating a potential role for these proteins as components of SlARF3-containing complexes.

### 2.5. Analysis of Protein–Protein Interactions for SlARF3 by Yeast Two-Hybrid (Y2H) and Bimolecular Fluorescence Complementation (BiFC)

**Figure 4:**
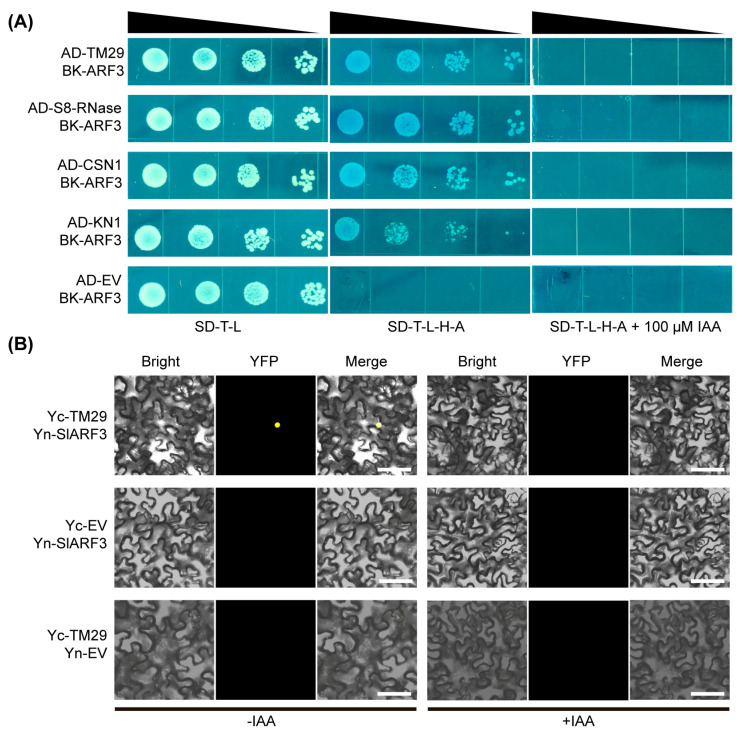
Y2H and BiFC assay confirming the interaction between SlARF3 and candidate proteins. (**A**) Yeast cells were co-transformed with the bait construct BK-SlARF3 and the prey construct AD-interactor and selected on SD-T-L medium. Protein–protein interaction was assessed on SD-T-L-H-A medium (±IAA) supplemented with 50 mg/L X-α-Gal; growth and blue coloration indicate a positive interaction. Co-transformation with empty prey and bait vectors served as the negative control. (**B**) SlARF3-TM29 interaction validated by BiFC under IAA treatment. YFP signal in *N. benthamiana* leaves co-expressing Yn-SlARF3 and Yc-TM29, with versus without 100 μM IAA treatment. YFP fluorescence signals were captured by confocal laser scanning microscopy at 48 h post-infiltration. Bars, 50 μm.

To further elucidate the interactions of SlARF3, the full-length CDSs of these candidates were amplified and subcloned into the pGADT7 vector for Y2H. Each resulting construct was subsequently co-transformed with BK-SlARF3 into the yeast strain AH109. All four co-transformants exhibited robust growth on SD-T-L medium as well as on SD-T-L-H-A medium supplemented with X-α-Gal, indicating positive protein–protein interactions. In contrast, their growth was completely abolished on SD-T-L-H-A medium containing IAA, demonstrating that the interaction between SlARF3 and these candidate partners is negatively regulated by auxin and thus auxin-sensitive (Figure 4A).

The functional similarity between TM29 and SlARFs in parthenocarpy prompted us to further validate the protein–protein interaction between SlARF3 and TM29 [25,26,27]. BiFC plasmids Yn-SlARF3 and Yc-TM29 were constructed by fusing the SlARF3 and TM29 CDSs to the N- and C-terminal fragments of YFP, respectively. The BiFC assay demonstrated that SlARF3 interacts with TM29 specifically within the nucleus. Furthermore, exogenous application of IAA markedly attenuated this interaction (Figure 4B), consistent with the results obtained from the Y2H analysis (Figure 4A). Collectively, the Y2H and BiFC assays provide convergent evidence that SlARF3 and TM29 engage in an auxin-sensitive protein–protein interaction mechanism.

### 2.6. Expression Patterns of SlARF3 and Its Interactors in Response to IAA

To further assess whether *SlARF3* and its interacting proteins are responsive to auxin, three-week-old tomato seedlings were treated with exogenous IAA by foliar spraying. Leaf tissues were harvested at 0 h, 6 h and 12 h post-treatment for quantitative real-time PCR (qRT-PCR) analysis, with the 0-h samples serving as the baseline. Members of the *GRETCHEN HAGEN3* (*GH3*) gene family encode enzymes that catalyze the conjugation of IAA to amino acids [28]; thus, changes in *SlGH3.4* transcript abundance can serve as a reliable indicator of auxin responsiveness. qRT-PCR analysis revealed that *SlGH3.4* expression was significantly up-regulated at both 6 h and 12 h, confirming that the auxin treatment effectively activated IAA signaling in the tomato seedlings at these time points.

**Figure 5:**
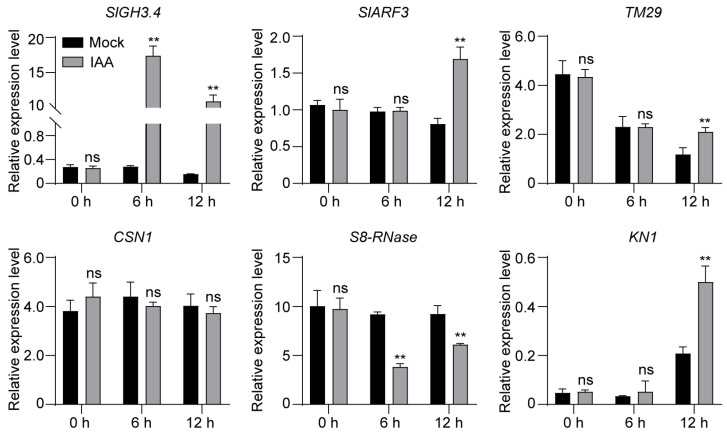
Analysis of relative expression levels of *SlARF3* and interactors in response to IAA treatment. Leaf samples from three-week tomato seedlings treated with IAA were collected at 0 h, 6 h and 12 h time points for qRT-PCR analysis. Gray and black bars represent samples treated with exogenous IAA and mock-treated controls (water), respectively. Data are presented as means ± SD (n = 3). **, *p* < 0.01 (Student’s *t*-test). ns, not significant.

Under these conditions, *SlARF3*, *TM29*, and *KN1* transcripts were positively regulated by IAA at 12 h. In contrast, *S8-RNase* expression was down-regulated at both 6 h and 12 h following IAA application. Distinct from these genes, *CSN1* did not exhibit a detectable transcriptional response to IAA treatment at either time point. Collectively, these results demonstrate that the transcript levels of *SlARF3*, *TM29*, *S8-RNase*, and *KN1* are responsive to IAA, whereas *CSN1* expression is not altered by treatment (Figure 5).

### 2.7. SlARF3 May Regulate Parthenocarpy in Tomato Through an Auxin-Sensitive Interaction with TM29

**Figure 6:**
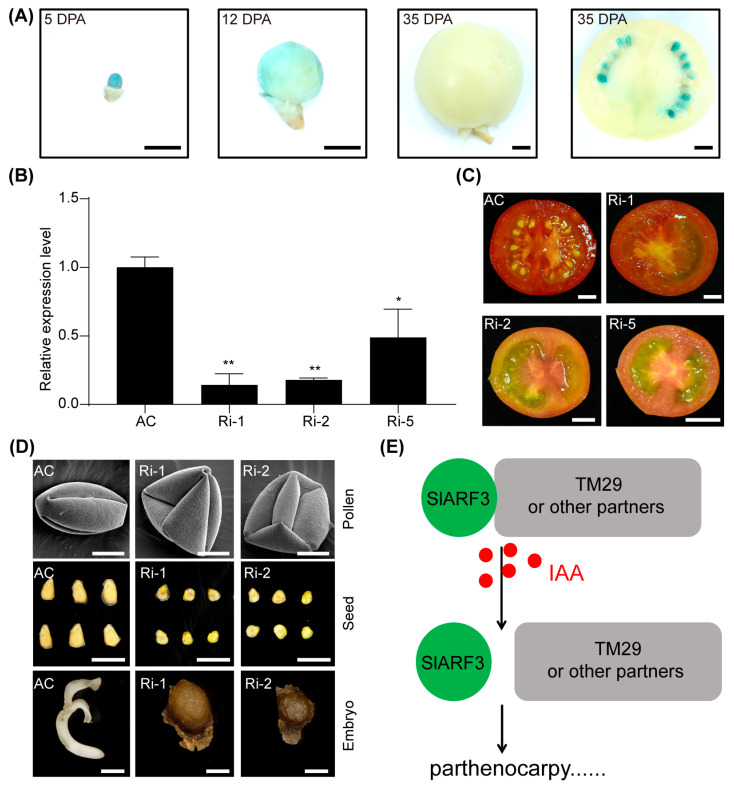
SlARF3 functional characterization in parthenocarpy. (**A**) Visualization of *SlARF3* expression at different developmental stages of ovaries. Ovaries from *ProSlARF3*: *GUS* transgenic line at 5, 12 and 35 days post anthesis (DPA) were incubated in GUS staining solution for 24 h and subsequently decolorized. The distribution and intensity of GUS staining reflect the spatial expression and relative abundance of *SlARF3*. Bars, 0.25 cm. (**B**) Analysis of silencing efficiency in *SlARF3*-RNAi lines. Flowers from transgenic lines were collected to determine the relative expression level of *SlARF3*, with *ACTIN* used as the internal reference. Data are presented as means ± SD (n = 3). *, *p* < 0.05; **, *p* < 0.01 (Student’s *t*-test). (**C**) Phenotypic analysis of parthenocarpy in *SlARF3*-RNAi lines. AC, Ri-1, Ri-2, and Ri-5 represent the wild-type Ailsa Craig (AC), *SlARF3*-RNAi-1, *SlARF3*-RNAi-2, and *SlARF3*-RNAi-5, respectively. Bars, 1 cm. (**D**) Observations included pollen morphology (bars, 10 μm), seed appearance (bars, 0.5 cm) and embryonic structure (bars, 1 mm) in the RNAi lines. Wild-type pollen was used to pollinate the *SlARF3*-RNAi lines, yielding seeds and embryos. (**E**) Schematic model illustrating the mechanism by which SlARF3 interacts with TM29 to mediate parthenocarpy. IAA disrupts the SlARF3-TM29 interaction, and exogenous auxin application could induce parthenocarpy phenotypes comparable to those resulting from silencing of either *SlARF3* or *TM29*.

To elucidate the physiological role of *SlARF3* during ovary development in tomato, we generated a GUS reporter construct driven by the *SlARF3* promoter. The construct was introduced into the tomato cultivar Ailsa Craig (AC) to establish a stable transgenic GUS reporter line. During fruit ripening, *SlARF3* expression progressively declined but still remained detectable in seeds, implying a potential function in ovary and seed development (Figure 6A).

Three lines exhibiting effective RNA interference of *SlARF3* were successfully identified, designated as *SlARF3*-RNAi-1 (Ri-1), *SlARF3*-RNAi-2 (Ri-2), and *SlARF3*-RNAi-5 (Ri-5), with interference efficiencies of 86%, 83%, and 51%, respectively (Figure 6B). To elucidate whether SlARF3 affects the expression of *TM29*, *S8-RNase*, *CSN1*, and *KN1*, the expression levels of these genes were analyzed in three independent *SlARF3*-RNAi lines. Their expression was down-regulated to varying degrees across these RNAi lines, indicating that the reduction in *SlARF3* expression may trigger feedback regulatory mechanisms, leading to decreased transcript levels of these interactors (Figure S5).

Phenotypic analysis of the *SlARF3*-RNAi knockdown lines revealed a suite of developmental alterations, including the anticipated and consistent alteration in leaf trichome formation (Figure S6A), in agreement with previous findings [20]. In addition, close examination showed that pistils of *SlARF3*-RNAi plants were shorter and thicker than those of the wild type, whereas stamen length remained nearly unchanged (Figure S6B). Finally, fruits from *SlARF3*-RNAi plants developed a characteristic pointed tip (Figure S6C) and conspicuously lacked an activated abscission zone at the pedicel (Figure S6D).

Our studies have demonstrated a nuclear interaction between SlARF3 and TM29 (Figure 4). In addition, two direct interaction interfaces between them were predicted by in silico analysis (Figure S7). TM29 is a MADS-box transcription factor that shares high sequence homology with the Arabidopsis SEPALLATA1, SEPALLATA2, and SEPALLATA3 proteins. It is predominantly expressed in tomato floral organs and young fruits. Notably, suppression of *TM29* expression in tomato results in a parthenocarpic phenotype [25]. Concurrent with these analyses, the *SlARF3*-RNAi lines displayed severe pollen abortion, consequently failing to achieve successful pollination (Figure 6D). However, these lines developed parthenocarpic fruits (Figure 6C), a phenotype identical to that observed in *TM29*-silenced plants [25]. Nonetheless, repeated attempts at cross-pollination of the RNAi lines with wild-type pollen yielded exclusively smaller, morphologically aberrant seeds that subsequently underwent embryo abortion (Figure 6D). These observations strongly indicate that SlARF3 is essential for embryo viability and fertility.

Based on these findings, a mechanistic model is proposed for parthenocarpy: auxin disrupts the SlARF3-TM29 interaction (or that with other partners), thereby inactivating the complex and triggering parthenocarpic phenotype. Future efforts should therefore focus on identifying these additional interactors, which are pivotal for elucidating the complete pathway (Figure 6E).

## 3. Discussion

As a member of the atypical AUXIN RESPONSE FACTOR (ARF) transcription factors that lack the PB1 domain, ARF3 mediates auxin signaling through auxin-responsive protein–protein interactions rather than the canonical PB1-dependent mechanism. In *Arabidopsis thaliana*, multiple genes have been identified that interact with ARF3 to facilitate its regulatory activities. *INDEHISCENT* (*IND*) encodes a basic helix–loop–helix (bHLH) transcription factor that is indispensable for the specification of the valve margin in *A. thaliana*. The valve margin represents a specialized tissue layer that controls fruit dehiscence and thereby facilitates seed dispersal. ARF3 has been shown to physically interact with IND in the nucleus in an IAA-sensitive manner, and this interaction is proposed to co-regulate the transcription of downstream target genes during pistil development. In addition, this study has demonstrated an interaction between AtARF3 and ABERRANT TESTA SHAPE (ATS) during ovule development, and this interaction is likewise modulated by IAA [29]. Further supporting this regulatory model, under low auxin conditions, AtARF3 forms a transcriptional corepressor complex with TOPLESS (TPL) and HDA19 to repress target genes via histone deacetylation. However, upon elevated auxin, direct hormone binding to AtARF3 triggers complex dissociation, leading to accumulated histone acetylation and consequent derepression of its target genes [14].

A Y2H screen using AtARF3 as bait identified several interacting transcription factors, including three HOMEOBOX proteins—REPLUMLESS (RPL), KNOTTED-LIKE FROM ARABIDOPSIS THALIANA1 (KNAT1), and KNAT3—as well as two TCP-family transcription factors and one AP2-domain protein [29]. In addition, a protein exhibiting high sequence homology to Arabidopsis KNAT1 was identified as a KNOTTED1-LIKE HOMEOBOX (KN1) in this study. This *KN1* homolog displays elevated transcript accumulation in the pedicel abscission zone. Moreover, silencing of *KN1* in tomato caused a significant decrease in floral abscission efficiency [30]. Consistently, *SlARF3*-RNAi lines exhibited reduced abscission in the pedicel abscission zone (Figure S6D). These observations support a model in which SlARF3 may perceive or transduce auxin signals via physical interaction with KN1 to modulate the floral abscission process. Nonetheless, the detailed molecular mechanism underlying this regulatory pathway remains to be elucidated.

In the gametophytic self-incompatibility (GSI) system that is widespread among angiosperms, S-RNase functions as the stylar S-determinant and is indispensable for the execution of this recognition mechanism [31]. Unexpectedly, a physical interaction between SlARF3 and S8-RNase was observed (Figure 4A). In Brassicaceae, the auxin response factor *ARF3* has been demonstrated to serve as a central regulator linking self-incompatibility (SI) with pistil development. *AtARF3* is expressed in the stylar vasculature and, through non-cell-autonomous signaling, enhances the SI response in the stigma while concurrently suppressing its auxin responsiveness. This regulatory framework integrates genetic self/non-self recognition with developmental programs, thereby facilitating outcrossing [18]. Taken together, these observations suggest that, in tomato, ARF3 may influence self-compatibility by integrating auxin signaling with S8-RNase-mediated processes. The exact molecular mechanism underlying this coordination remains to be elucidated and will require further experimental investigation.

As the central subunit of the COP9 signalosome (CSN), CSN1 constitutes a key regulatory element within the auxin signaling pathway through its tight control of ubiquitin–proteasome system activity, the principal cellular machinery responsible for targeted protein degradation. In particular, the CSN complex directly associates with and stabilizes the SCF^TIR1^ E3 ubiquitin ligase complex. This interaction is essential for facilitating the timely proteasomal degradation of AUX/IAA transcriptional repressor proteins, thereby enabling precise spatiotemporal regulation of auxin signal transduction [32]. Consistent with this regulatory framework, our data demonstrate an IAA-dependent interaction between SlARF3 and CSN1 (Figure 4A). These observations lead us to propose that SlARF3 may initiate a distinct auxin signaling cascade by directly perceiving the hormone through its interaction with CSN1. Elucidating the dynamics, composition, and regulatory properties of this protein complex will therefore be essential for a comprehensive understanding of this putative signaling pathway.

Parthenocarpy in tomato refers to the phenomenon wherein ovaries develop into seedless fruits without fertilization. The central regulatory mechanism underlying this process is orchestrated by the auxin signaling pathway, which encompasses a series of precisely coordinated events, including localized auxin biosynthesis within ovules, followed by its perception and subsequent signal transduction [33,34,35]. In previous studies, numerous auxin signaling-related genes have been demonstrated to be involved in the regulation of tomato parthenocarpy. Functional impairment of the auxin response factors ARF2, ARF5, and ARF7 results in clear parthenocarpy in tomato, indicating that these *ARF* family members function as negative regulators of fruit set [26,27,36]. Furthermore, loss-of-function mutations in the HD-Zip III transcription factor *HOMEOBOX-LEUCINE ZIPPER PROTEIN 15 A* (*HB15A*) confer a pronounced capacity for parthenocarpic fruit development, whereas loss of function in the two MADS-box transcription factors *AGAMOUS-LIKE 6* (*AGL6*) and *TM29* similarly results in the induction of parthenocarpy in tomato [25,37,38]. Our experimental results indicate that SlARF3 interacts with TM29 (Figure 4), and knockdown of *SlARF3* expression in tomato can also induce parthenocarpy (Figure 6C), suggesting a critical role of the SlARF3-TM29 module in tomato parthenocarpy. More importantly, auxin can disrupt this interaction, which may partially elucidate the molecular mechanism underlying auxin-induced parthenocarpy in tomato and thus warrants further in-depth theoretical and experimental investigation (Figure 6E).

The Y2H system is optimal for identifying SlARF3 interactors, as it circumvents endogenous IAA interference. By this approach, we performed a systematic interaction screen of *SlARF3*, yielding critical insights into how *SlARF3* modulates auxin signaling through auxin-responsive protein–protein interactions. These findings substantially provide a direct entry point for future studies to elucidate the specific roles of SlARF3 in governing tomato developmental processes.

In conclusion, this study uncovers a SlARF3-centered interaction network and proposes a novel conceptual framework for understanding non-canonical auxin signaling pathways during tomato development. Using Y2H screen, 118 candidate proteins were identified interacting with SlARF3, all of which, intriguingly, were disrupted by IAA treatment. Leveraging the parthenocarpic phenotype of *SlARF3*-RNAi lines, we further confirmed through Y2H and BiFC assays that SlARF3 physically interacts with the key regulator TM29 to modulate parthenocarpy in tomato. These findings hold great significance, as these interacting proteins offer valuable clues for further clarifying the functional role of SlARF3 in tomato biology.

## 4. Materials and Methods

### 4.1. Plant Materials and Growth Conditions

The plant materials used in this study, including the tomato (*Solanum lycopersicum*) cultivar Ailsa Craig (AC), *Nicotiana benthamiana*, as well as the transgenic *ProSlARF3: GUS* reporter line and the *SlARF3*-RNAi knockdown lines, were cultivated in a controlled environment growth chamber. The chamber conditions were maintained at a temperature of 24 °C and a relative humidity of 40%. Plants were subjected to a photoperiod of 16 h of light and 8 h of darkness, with a photosynthetic photon flux density (PPFD) of 600 µmol m^−2^ s^−1^ provided during the light phase.

### 4.2. RNA Extraction and Yeast Library Construction

Root, stem, leaf, flower, young fruit, and mature fruit tissues were collected from eight-week-old AC plants, immediately frozen in liquid nitrogen, and ground into a fine powder. Total RNA was subsequently extracted from these powdered tissues using a commercial RNA extraction kit (Tiangen, Beijing, China) according to the manufacturer’s protocol. The extracted mRNA was enriched via oligo(dT) beads (Thermo Fisher Scientific, Waltham, MA, USA) and reverse-transcribed into complementary DNA (cDNA) (Vazyme, Nanjing, China). A cDNA library was then constructed by recombining the synthesized triptych-frame cDNA into the pGADT7 vector using a homologous recombination system (Vazyme, Nanjing, China). The resulting recombinant plasmids were introduced into *E. coli* TOP10 competent cells via electroporation. Following transformation, the library titer was determined, and the average insert size was verified. A large-scale plasmid preparation (Tiangen, Beijing, China) was performed from the validated library culture to obtain the final library plasmid stock, which was stored at −20 °C for subsequent use. The experimental procedures were conducted following the manufacturer’s protocol corresponding to each reagent.

### 4.3. Quantitative Real-Time PCR Analysis

Quantitative real-time PCR (qRT-PCR) assays were conducted using a Thermo Fisher QuantStudio 3 system. The thermal cycling protocol was configured in accordance with the specifications of the 2 × M5 FastSYBR Mixture (Mei5bio, Beijing, China). Tomato *ACTIN* (*Solyc11g005330*) served as the endogenous control for normalization [39]. Relative gene expression levels were determined using the 2^−ΔΔCT^ method [40].

### 4.4. Vector Construction

Gene sequences used for vector construction were obtained from the Solanaceae Genomics Network database (https://solgenomics.net/, accessed on 27 September 2024). To construct the BK-SlARF3 vector, the full-length coding sequence (CDS) of *SlARF3* was amplified using gene-specific primers and subsequently cloned into the pGBKT7 vector between the *Eco*RI and *Sal*I restriction sites via homologous recombination. For the generation of AD-TM29, AD-S8-RNase, AD-CSN1 and AD-KN1 vectors, the respective full-length CDS fragments were individually recombined into the pGADT7 vector between the *Eco*RI and *Bam*HI sites. The *ProSlARF3: GUS* reporter vector was assembled by inserting a 2.5 kb promoter fragment of the *SlARF3* gene into the PMV2 vector at the *Eco*RI site [41]. To create the *SlARF3*-RNAi vector, a 400 bp gene-specific fragment was directionally cloned in both sense and antisense orientations into the pHELLSGATE8 vector, utilizing the *Xho*I and *Xba*I restriction sites, respectively. The construction of all vectors was performed using homologous recombination technology (Vazyme, Nanjing, China). All primer sequences used for these constructions are listed in Supplementary Table S2.

### 4.5. Transient Expression in Tobacco Leaves

The auxin-responsive *DR5* promoter was cloned into the reporter vector pMV2-GUS, a derivative of pHELLSGATE8 [41]. For effector construction, *SlARF3* CDS was inserted into pHELLSGATE8 to be driven by the cauliflower mosaic virus (CaMV) 35S promoter, with the empty vector used as a negative control. Reporter and effector constructs were co-expressed into young tobacco leaves via agroinfiltration. After 48 h, leaves were collected for GUS staining and qRT-PCR.

### 4.6. GUS Staining

For GUS staining, tissues were fully immersed in staining solution (containing 10 mM EDTA disodium salt, 100 mM sodium phosphate buffer (pH 7.0), 10 mM Na_2_EDTA, 1 mM potassium ferrocyanide, 1 mM potassium ferricyanide, 0.1% (*v*/*v*) Triton X-100, 0.1% N-laurylsarcosine, and 0.5 mg/mL X-gluc). Following vacuum infiltration (0.08 MPa, 5 min) as needed and overnight incubation at 37 °C under dark conditions, samples were cleared in 75% ethanol at 70 °C for 3 h. For long-term preservation, samples were kept in 75% ethanol at room temperature.

### 4.7. SlARF3-RNAi Lines Phenotypic Analysis

To identify the phenotypes of leaf trichomes and styles in *SlARF3-RNAi* lines. Leaflets from the third node below the apical meristem and flowers in full bloom of 7-week-old tomato plants were determined using a Nikon SMZ25 stereomicroscope (Nikon, Tokyo, Japan). Green maturation stage fruit phenotypes were determined by the Nikon 750 digital camera. The pollen structure at the full-bloom stage was analyzed using a Thermo Fisher Helios 5cx scanning electron microscope. Cryoscanning electron microscopy (cryo-SEM) was conducted as per prior methodology [42]. In brief, pollen from mature flowers was rapidly frozen in liquid nitrogen and subsequently gold-sputter-coated using the airlock interface of a Thermo Fisher Helios 5cx system for observation.

### 4.8. Exogenous Application of IAA to Tomato Seedlings

Three-week-old AC seedlings were treated with 50 μM IAA (Solarbio, Beijing, China) or distilled water (control) by spray application until runoff. Treatment was performed once, and leaf samples were collected at 0, 6 and 12 h post-treatment for RNA extraction and qRT-PCR analysis.

### 4.9. Y2H Library Screening and Y2H Assays

The yeast strain AH109 was employed for both library screening and pairwise interaction assays. The BK-SlARF3 bait construct was transformed into AH109 competent cells and plated on appropriate dropout medium. A single transformant colony was selected, inoculated into liquid dropout medium for propagation, and subsequently used to prepare competent cells harboring the bait plasmid. For the library screen, the prepared cDNA library plasmids were transformed into these BK-SlARF3-containing competent cells. The transformation mixture was plated onto SD-T-L-H-A medium and incubated at 30 °C for 4–6 d. Colonies that grew were subsequently re-streaked onto SD-T-L medium and onto SD-T-L-H-A medium supplemented with X-α-galactosidase (X-α-gal). Putative positive clones were subjected to colony PCR amplification using T7 and 3′ AD vector-specific primers (Table S2), followed by direct Sanger sequencing. The obtained sequences were aligned against the tomato genome database (https://solgenomics.net/, accessed on 10 September 2024) to identify genes encoding potential SlARF3-interacting proteins.

For the directed Y2H assays, BK-SlARF3 was co-transformed with individual prey constructs (AD-TM29, AD-KN1, AD-S8-RNase, AD-CSN1) into AH109 competent cells. Transformants were selected on SD-T-L medium and subsequently tested for interaction by streaking on SD-T-L-H-A medium. To assess auxin sensitivity, transformants were also plated on SD-T-L-H-A medium supplemented with 100 µM IAA. All plates were incubated at 30 °C for 3 days, and growth was documented.

### 4.10. Gene Ontology (GO) and Kyoto Encyclopedia of Genes and Genomes (KEGG) Enrichment

The annotation data required for GO and KEGG analyses were retrieved from the KEGG Orthology-Based Annotation System (KOBAS, version 2.0) [43], a publicly accessible platform that provides comprehensive functional annotation links between genes and pathways. The GO and KEGG enrichment maps were visualized with R (version 4.5.2).

### 4.11. Bimolecular Fluorescence Complementation (BiFC) Assays

BiFC assays were performed to investigate the protein–protein interactions in planta. The CDS of *SlARF3* was engineered into the cYFP vector, while the CDSs of *KN1*, *TM29*, *CSN1*, and *S8-RNase* were individually cloned into the nYFP vector [23]. These constructs were introduced into *Agrobacterium tumefaciens* strain GV3101 via heat shock transformation (WEIDI, Shanghai, China). The transformed bacteria were cultured on solid LB medium supplemented with appropriate antibiotics at 28 °C for 48 h. Single colonies were then selected for colony PCR verification using plasmid-specific primers. A positive clone confirmed by PCR was inoculated into liquid LB medium with antibiotics and incubated overnight at 28 °C with shaking at 220 rpm to obtain an active culture for subsequent experiments. The bacterial cells were harvested by centrifugation, resuspended in an infiltration buffer (10 mM MES, 10 mM MgCl_2_, 200 μM acetosyringone), and adjusted to an optical density (OD_600_) of 0.2. The suspensions were co-infiltrated into leaves of *Nicotiana benthamiana* plants. After 48 h of infiltration, YFP fluorescence was observed using a Nikon A1 confocal microscope (Nikon, Tokyo, Japan).

### 4.12. Subcellular Localization

The full-length CDSs of *SlARF3*, *KN1*, *CSN1*, *TM29*, and *S8-RNase* were individually fused in-frame to the C-terminus of *eGFP* in the pK7WGF2 vector via cloning at the *Bsr*GI restriction sites, utilizing Exnase II (Vazyme, Nanjing, China) for recombination. Subsequently, the constructed plasmid was introduced into *Agrobacterium tumefaciens* strain GV3101 via heat shock transformation (WEIDI, Shanghai, China). The preparation steps for the bacterial suspension prior to experimental use, including the adjustment of OD_600_, followed the identical procedure described in Section 4.11. Following a 48 h incubation period, eGFP fluorescence was assessed using a Nikon A1 confocal microscope (Nikon, Tokyo, Japan).

## Data Availability

The original contributions presented in this study are included in the article/Supplementary Materials. Further inquiries can be directed to the corresponding authors.

## Supplementary Materials

The following supporting information can be downloaded at: https://www.mdpi.com/article/10.3390/ijms27031227/s1.
