# Supplementary material for: A Noncanonical Auxin-Sensing Mechanism Uncovered by Screening the Auxin Response Factor 3 Interacting Proteins in Tomato
Source: Int J Mol Sci. 2026 Jan 26;27(3):1227. doi: 10.3390/ijms27031227 (PMC12897879; doi:10.3390/ijms27031227)
Supplement: Supplementary file 1 [file ijms-27-01227-s001.zip › ijms-4104906-supplementary.pdf]

Supplementary Material

A Noncanonical Auxin-Sensing Mechanism Uncovered by Screening the Auxin Response Factor 3 Interacting Proteins in tomato

Lin Wang, Xirong Yang, Sidratul Muntha, Liepeng Dong, Qingmin Xie, Taotao Wang, Chunmei Shi and Changxian Yang

Figure S1 Analysis of *SlARF3* relative expression in different tissues
Figure S2 Identification and analysis of insertion fragments from the secondary library
Figure S3 Identification of transactivation activity by SlARF3 and IAA toxicity in AH109
Figure S4 Identification of SlARF3 interactors
Figure S5 Analysis of relative expression levels of interactors in *SlARF3*-RNAi lines
Figure S6 Phenotypes of *SlARF3*-RNAi lines in tomato
Figure S7 Predicted interaction between SlARF3 and TM29 by AlphaFold
Supplementary Table S1 Putative interaction proteins of SlARF3 identified from the Y2H
Supplementary Table S2 A list of primers used in this study

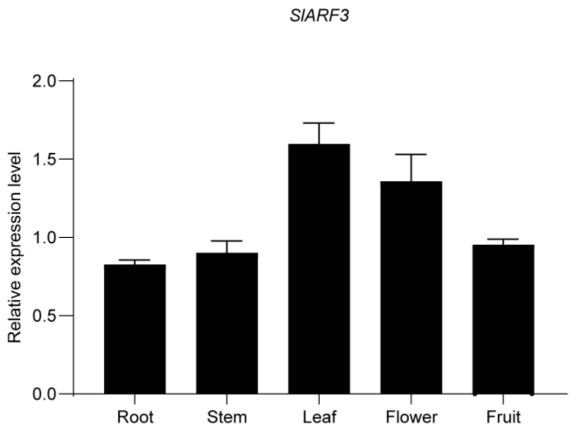

Figure S1. Analysis of *SlARF3* relative expression in different tissues. Data are presented as means ± SD (n = 3).

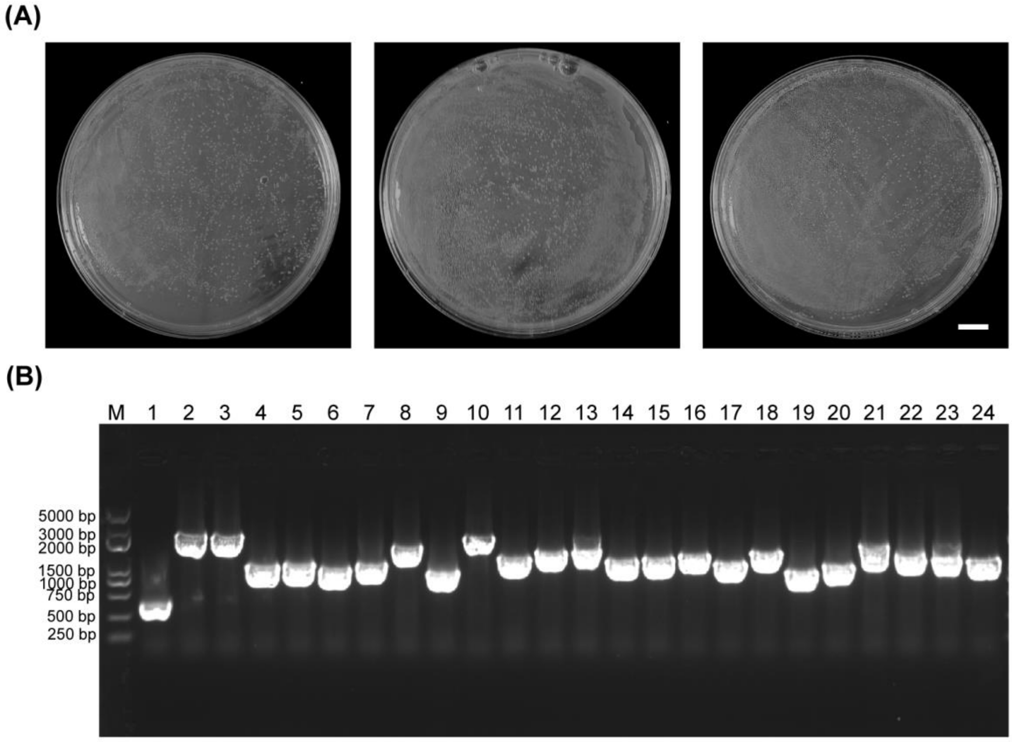

Figure S2. Identification and analysis of insertion fragments from the secondary library. (A) Identification of library cell density. Bar, 0.5 cm. (B) Identification of fragments from secondary library by PCR (lane 1-24) and Marker DL5000 (lane M).

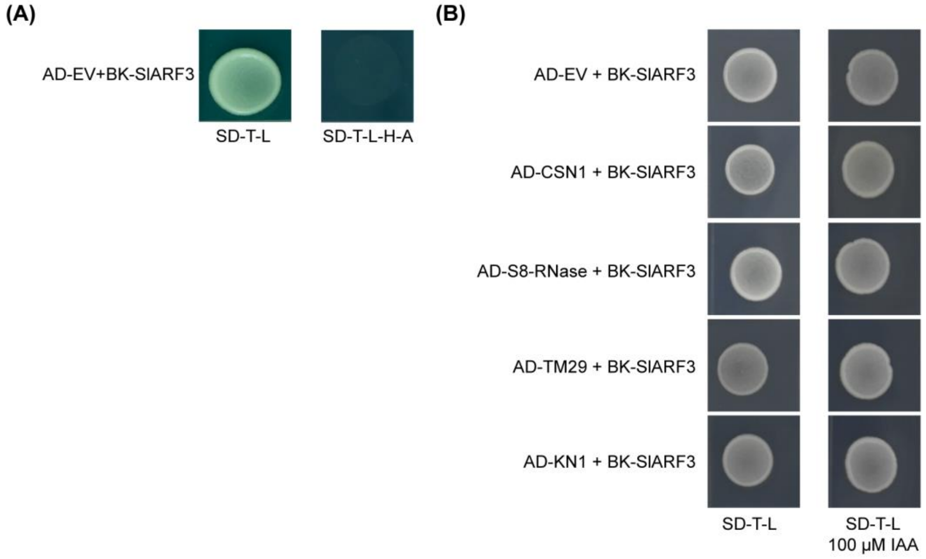

Figure S3. Identification of transactivation activity by SlARF3 and IAA toxicity in AH109
(A) Identification of transactivation activity by SlARF3. The BK-SlARF3 and pGADT7 (AD-EV) were co-expressed in AH109. (B) Identification of IAA toxicity in AH109. Yeast cells co-transformed with BK-SlARF3 (bait) and AD-interactor (prey) constructs were cultured on selection medium (SD-T-L ± IAA) and AD-EV served as negative control.

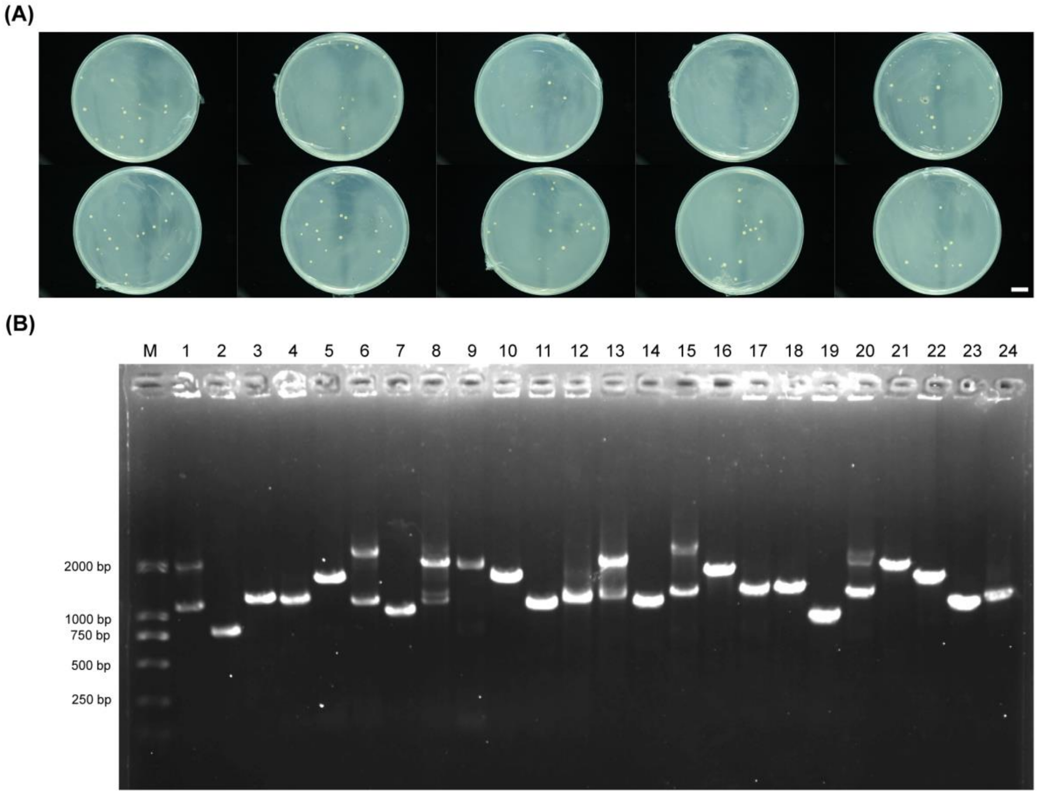

Figure S4. Identification of SlARF3 interactors. (A) Identification of interactors from the secondary library on SD-T-L-H-A. Bar, 2cm. (B) Identification of insertion fragments by PCR (lane 1-24) and Marker DL2000 (lane M).

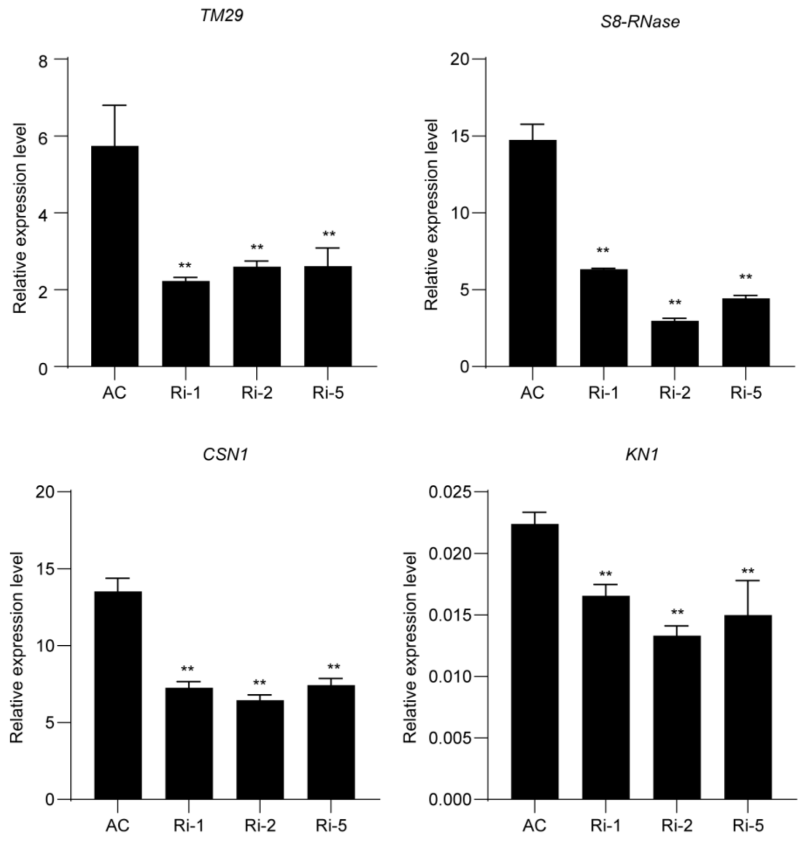

Figure S5. Analysis of relative expression levels of interactors in *SlARF3*-RNAi lines. Leaf samples from tomato plants for qRT-PCR analysis. Data are presented as means ± SD (n = 3). *, $P < 0.05$; **, $P < 0.01$ (Student's *t*-test).

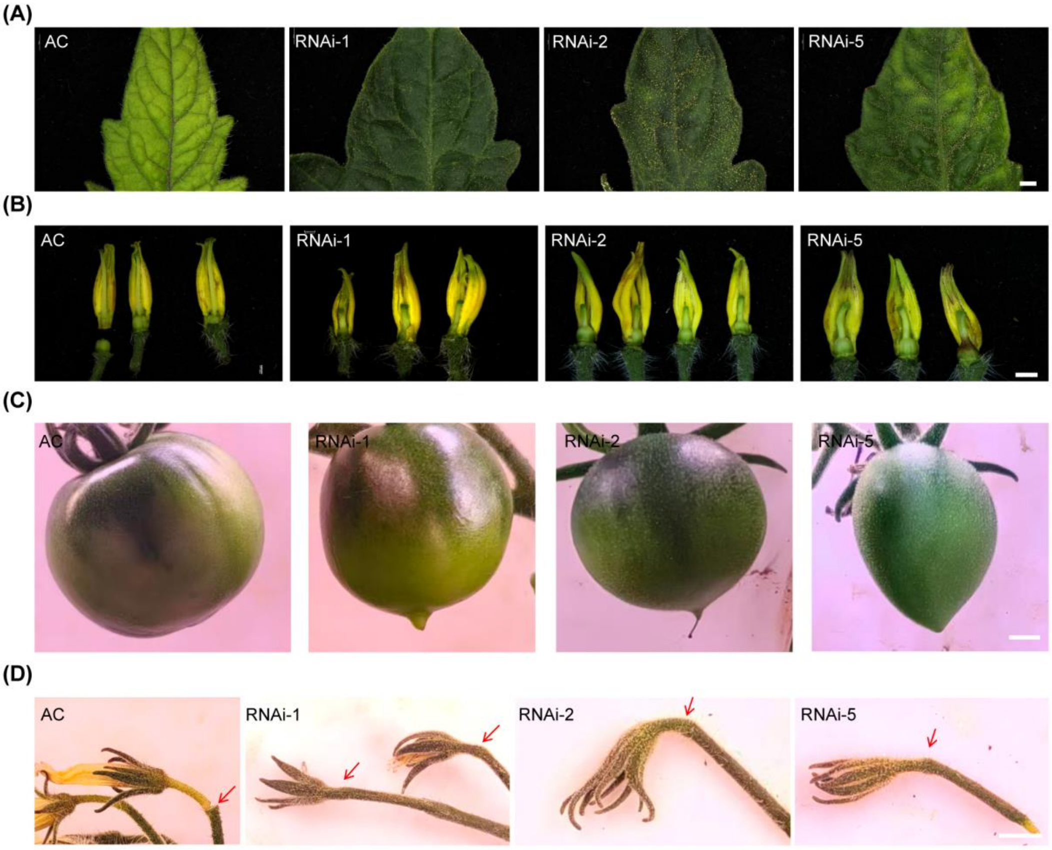

Figure S6. Phenotypes of *SlARF3*-RNAi lines in tomato. (A) AC and *SlARF3*-RNAi lines leaflets were imaged using a stereomicroscope. Leaflets were collected from the third fully expanded node below the apical meristem. Bar, 0.25 cm. (B) AC and *SlARF3*-RNAi lines flowers were imaged using a stereomicroscope. Samples collected at the full-bloom stage were prepared with petals removed and half of the stamens dissected away, followed by observation under a stereomicroscope. Bar, 0.25 cm. (C) AC and *SlARF3*-RNAi lines fruits were imaged using a digital camera. The fruits were photographed at the mature green stage. Bar, 0.5 cm. (D) AC and *SlARF3*-RNAi lines pedicel were imaged using a digital camera. Flower retention in *SlARF3*-RNAi lines versus abscission in AC due to presence or absence of an activated abscission zone. The red arrow indicates the abscission zone. Bar, 0.5 cm.

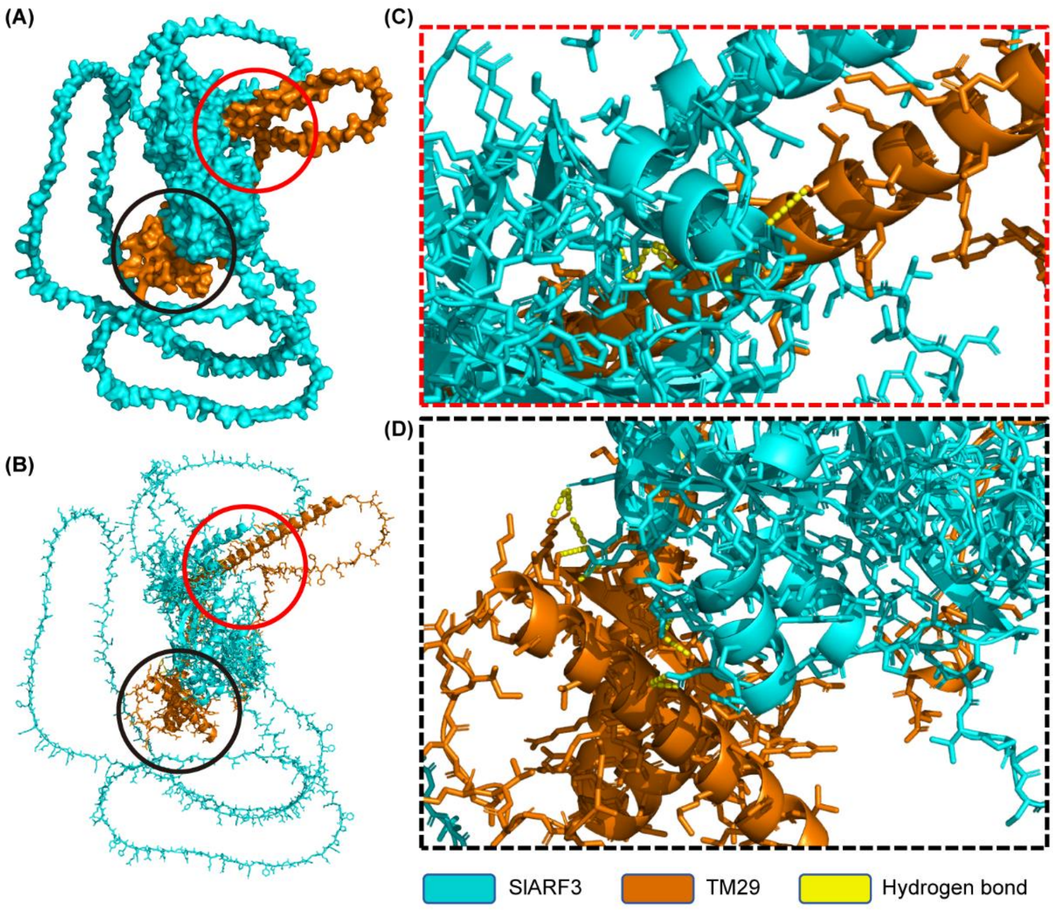

Figure S7. Predicted interaction between SlARF3 and TM29 by AlphaFold. (A) and (B) show the three-dimensional structure of the SlARF3–TM29 complex, while (C) and (D) detail their interaction interface. The structure of the SlARF3–TM29 complex was predicted using the online AlphaFold Server (https://alphafoldserver.com/) and visualized with PyMOL (https://www.pymol.org/).

Supplementary Table S1. Putative interaction proteins of SlARF3 identified from the Y2H library

Gene ID	Annotated Function	Frequency
Solyc01g005640	Uncharacterized plant-specific domain TIGR01589 family protein	1
Solyc01g008960	SlAGO4a	1
Solyc01g009780	LITAF-domain-containing protein	1
Solyc01g028900	1-deoxy-D-xylulose-5-phosphate synthase	1
Solyc01g067890	1-D-deoxyxylulose 5-phosphate synthase	1
Solyc01g073890	CHP-rich zinc finger protein-like	1
Solyc01g081060	Xyloglucan endotransglucosylase/hydrolase 14	1
Solyc01g091010	SlYABBY1a	2
Solyc01g091650	COP9 signalosome complex subunit 1	1
Solyc01g094010	CXE carboxylesterase	1
Solyc01g094660	Receptor-like protein kinase	1
Solyc01g095490	Ubiquitin-conjugating enzyme E2 8	1
Solyc01g096570	Exonuclease family protein expressed	1
Solyc01g096780	Sperm-associated SUN domain protein	1
Solyc01g099090	Beta-mannosidase	1
Solyc01g099790	AT3G05545 protein (Fragment)	1
Solyc01g100720	Importin alpha-1b subunit	1
Solyc01g104680	Ran protein/TC4 protein ran2a	1
Solyc01g106320	Octicosapeptide/Phox/Bem1p domain-containing protein	1
Solyc01g110430	UNknown	1
Solyc01g111520	Synaptotagmin	1
Solyc02g068500	Thioredoxin o	2
Solyc02g071610	GDSL esterase/lipase At5g45670	1
Solyc02g076710	Cathepsin B-like cysteine proteinase	1
Solyc02g077040	phytophthora-inhibited protease 1	1
Solyc02g078400	Allantoinase	1
Solyc02g082200	Glutaredoxin	4
Solyc02g082760	ethylene-responsive catalase	1
Solyc02g085950	cell wall protein X77373	1
Solyc02g089200	TM29	1
Solyc02g089800	Nuclear matrix constituent protein 1-like	1
Solyc02g093470	Lysine ketoglutarate reductase trans-splicing related 1	1
Solyc03g007320	Polypyrimidine tract-binding protein 1-like	1
Solyc03g025600	Pectinacetylesterase like protein	1
Solyc03g034220	Tomato RuBP carboxylase small subunit	9
Solyc03g044150	Subtilisin-like protease	1
Solyc03g078400	actin	1
Solyc03g095710	Alpha-amylase	1

Gene ID	Annotated Function	Frequency
Solyc03g095900	E8 protein homolog	1
Solyc03g098440	Small glutamine-rich tetratricopeptide repeat-containing protein A	1
Solyc03g111810	Sieve element-occluding protein 3	1
Solyc03g116230	Agglutinin isolectin I	1
Solyc03g119300	CHY zinc finger containing protein	1
Solyc03g121280	Os03g0731050 protein	1
Solyc04g005340	Alpha-1 4-glucan protein synthase	1
Solyc04g015020	Proline-rich protein	1
Solyc04g077210	Knotted 1	1
Solyc04g078110	serine protease SBT1	1
Solyc05g009150	CHY zinc finger containing protein	1
Solyc05g010420	S-adenosylmethionine decarboxylase proenzyme	1
Solyc05g045670	Glucose-6-phosphate/phosphate translocator 2	1
Solyc05g056270	Isocitrate lyase	1
Solyc06g006080	Phosphomethylpyrimidine synthase	2
Solyc06g009970	Elongation factor 1-alpha	1
Solyc06g034020	Peptide methionine sulfoxide reductase msrA	1
Solyc06g043170	Actin family protein	1
Solyc06g052040	Calcium-binding EF hand family protein	1
Solyc06g054450	UPF0454 protein C12orf49 homolog	1
Solyc06g073960	Serine/threonine-protein phosphatase	1
Solyc06g074710	Hydroxycinnamoyl CoA shikimate/quinate hydroxycinnamoyltransferase-like protein	1
Solyc06g076510	Phosphoserine phosphatase	1
Solyc07g006570	S8-RNase	1
Solyc07g007370	NEDD8 ultimate buster 1	1
Solyc07g021020	Glyoxal oxidase-like	1
Solyc07g041920	Cathepsin L-like cysteine proteinase	1
Solyc07g054540	Lipoyl synthase	1
Solyc07g055050	ATP synthase I-like protein	1
Solyc07g055700	Solute carrier family 35 member C2	1
Solyc07g063600	Chlorophyll a-b binding protein 13	1
Solyc07g063650	Ubiquitin carboxyl-terminal hydrolase	1
Solyc07g066150	Photosystem I reaction center subunit V	1
Solyc07g066650	DCN1-like protein 2	1
Solyc08g005470	Cell division protein kinase 7	1
Solyc08g006620	Tsi1-interacting protein TSIP1	1
Solyc08g008460/57	Kinesin light chain	1
Solyc08g048550	Protease Do-like (S2 serine-type protease)	1

Gene ID	Annotated Function	Frequency
Solyc08g061000	ATP-dependent RNA helicase	1
Solyc08g062970	Glutaredoxin	1
Solyc08g066450	Unknown Protein	1
Solyc08g075700	60S ribosomal protein L13	1
Solyc08g076970	Acetylornithine deacetylase or succinyl-diaminopimelate desuccinylase	1
Solyc08g082190	Unknown Protein	1
Solyc09g007490	Cell number regulator 8	1
Solyc09g008290	Thioredoxin family protein	1
Solyc09g009260	fructose-1,6-bisphosphate aldolase	1
Solyc09g011880	LOC556397 protein	1
Solyc09g061290	WD-40 repeat family protein	1
Solyc09g065330	40S ribosomal protein S24	1
Solyc09g066290	Kinesin light chain-like	1
Solyc09g075430	Ribosomal protein L19	1
Solyc09g082340	Vicilin-like protein	2
Solyc09g090150	Legumin 11S-globulin	1
Solyc09g092380	S-adenosyl-l-homocysteine hydrolase	1
Solyc09g098290	LRR receptor-like serine/threonine-protein kinase FEI 1	1
Solyc10g006040	Serine acetyltransferase	1
Solyc10g009220	Polyadenylate-binding protein 4-like	1
Solyc10g012080	MRNA clone RAFL21-79-C21	1
Solyc10g054440	Arginine decarboxylase	1
Solyc10g074580	Aminoacylase ACY1 and related metalloexopeptidases	1
Solyc10g075150	Non-specific lipid-transfer protein	1
Solyc10g078590	Unknown Protein	2
Solyc10g083440	UDP flavonoid 3-O-glucosyltransferase	1
Solyc10g084260	Cell number regulator 8	1
Solyc10g085200	Acyl-CoA dehydrogenase	2
Solyc11g066360	Unknown Protein	1
Solyc11g066870	Genomic DNA chromosome 5 P1 clone MDJ22	1
Solyc11g069430	SlPIP2.6	1
Solyc11g072660	Receptor protein kinase-like protein	1
Solyc12g009000	Os07g0175100 protein	1
Solyc12g014380	glucose-6-phosphate isomerase	1
Solyc12g019700	UNknow Protein	1
Solyc12g019860	CCR4-NOT transcription complex subunit 3	1
Solyc12g044600	NADP-malic enzyme	1
Solyc12g056210	MRNA clone RAFL21-79-C21	1
Solyc12g088670	cysteine-type endopeptidase activity	3

Gene ID	Annotated Function	Frequency
Solyc12g094620	catalase	1
Solyc12g098690	WD-repeat protein-like	1
Solyc12g099000	S-adenosylmethionine synthase	1

Supplementary Table S2. A list of primers used in this study

Primer name	Sequence (5'-3')	Vector	Usage
BK-ARF3-ECORI-F	TGCATATGGCCATGGAGGCCGAATTCA TGATGTGTGGACTTATTGATCTG	pGBKT7	Y2H assay
BK-ARF3-SalI-R	TAGTTATGCGGCCGCTGCAGGTCGACC TACAGAGCAATATCAAGAAGCACATC	pGBKT7	Y2H assay
AD-TM29-ECORI-F	TGGAGGCCAGTGAATTCATGGGTAGAG GAAGAGTTGAGC	pGADT7	Y2H assay
AD-TM29-BamHI-R	CAGCTCGAGCTCGATGGATCCTCACAG CATCCAACCAGGTATC	pGADT7	Y2H assay
AD-S8-RNase-ECORI-F	TGGAGGCCAGTGAATTCATGAAGAAAC TTGTTAACTTCTTTCTTCTTG	pGADT7	Y2H assay
AD-S8-RNase-BamHI-R	CAGCTCGAGCTCGATGGATCCTTACTG AGGTTTTGGATGAGGTAGC	pGADT7	Y2H assay
AD-CSN1-ECORI-F	TGGAGGCCAGTGAATTCATGGAGCCTG ACGAGGATTTAG	pGADT7	Y2H assay
NYFP-ARF3-BamHI-F	GATTTCTGAGGAGGATCTTGGATCCAT GATGTGTGGACTTATTGATCTG	NYFP	BiFC
NYFP-ARF3-SalI-R	GGGTACCGAATTCACTAGTGTCGACCT ACAGAGCAATATCAAGAAGCACATC	NYFP	BiFC
CYFP-TM29-BamHI-F	CGATGTTCCAGATTACGCTGGATCCAT GGGTAGAGGAAGAGTTGAGC	CYFP	BiFC
CYFP-TM29-SalI-R	GGGTACCGAATTCACTAGTGTCGACTC ACAGCATCCAACCAGGTATC	CYFP	BiFC
PK7-ARF3-BsrGI-F	CTCTCGGCATGGACGAGCTGTACAAGA TGATGTGTGGACTTATTGATC	pK7WGF2	Subcellular localization
PK7-ARF3-BsrGI-R	ATATCACCACTTTGTACACTACAGAGC AATATCAAGAAGC	pK7WGF2	Subcellular localization
PK7-TM29-BsrGI-F	CTCTCGGCATGGACGAGCTGTACAAGA TGGGTAGAGGAAGAGTTGAGC	pK7WGF2	Subcellular localization
PK7-TM29-BsrGI-R	ATATCACCACTTTGTACATCACAGCATC CAACCAGGTATC	pK7WGF2	Subcellular localization
PK7-S8-RNase-BsrGI-F	CTCTCGGCATGGACGAGCTGTACAAGA TGAAGAAACTTGTTAACTTCTTTCTTCT TG	pK7WGF2	Subcellular localization
PK7-S8-RNase-BsrGI-R	ATATCACCACTTTGTACATTACTGAGGT TTTGGATGAGGTAGC	pK7WGF2	Subcellular localization
PK7-CSN1-BsrGI-F	CTCTCGGCATGGACGAGCTGTACAAGA TGGAGCCTGACGAGGATTTAG	pK7WGF2	Subcellular localization
PK7-CSN1-BsrGI-R	ATATCACCACTTTGTACATCAATGTTTT CTTGACGTTCTAGCAATG	pK7WGF2	Subcellular localization

Primer name	Sequence (5'-3')	Vector	Usage
PK7-KN1-BsrGI-F	CTCTCGGCATGGACGAGCTGTACAAGATGGAGAATAATAATTATAATAATCATGTGTC	pK7WGF2	Subcellular localization
PK7-KN1-BsrGI-R	ATATCACCACTTTGTACATTACTGACCCAAACGAAAAGGG	pK7WGF2	Subcellular localization
ARF3-PMV2-SacI-F	TGCATCCAACGCGTTGGGAGCTCAAAACGGGTTGAAATTGGAG	PMV2	GUS effector
ARF3-PMV2-XhoI-R	GCCTTCGCCATTCTAGACTCGAGCTTTAAACCCTAAGATTAAACAGAGAG	PMV2	GUS effector
ARF3-RNAi-XhoI-F	TTTCATTTGGAGAGGACACGCTCGAGGTATCTCTCATCTGGTTCACATACCG	pHELLSGATE 8	RNA interference
ARF3-RNAi-XhoI-R	ATATCTCATTAAAGCAGGACTCTAGAGTATCTCTCATCTGGTTCACATACCG	pHELLSGATE 8	RNA interference
ARF3-RNAi-XbaI-F	AAATCGATAAGCTTGGATCCTCTAGACAGAGCAATATCAAGAAGCACATCTC	pHELLSGATE 8	RNA interference
ARF3-RNAi-XbaI-R	ATATCTCATTAAAGCAGGACTCTAGAGTATCTCTCATCTGGTTCACATACCG	pHELLSGATE 8	RNA interference
Q-actin-F	GTCCTCTTCCAGCCATCCA		qPCR
Q-actin-R	ACCACTGAGCACAATGTTACCG		qPCR
Q-SlGH3.4-F	GACAAGGCCATACGATCCATAC		qPCR
Q-SlGH3.4-R	GTATTCCCAAATGCTTTGTGGC		qPCR
Q-ARF3-F	TTGGGACACAGTCGTCATTC		qPCR
Q-ARF3-R	TCCGCTCTAGTTGCACTTATTT		qPCR
Q-TM29-F	AGATGAGTTGGGGCCTCTGA		qPCR
Q-TM29-R	TGAGACTTTGCTCACCACCA		qPCR
Q-S8-RNase-F	GAGGAACCACACCACAAGTAT		qPCR
Q-S8-RNase-R	AGACAAGCTATCACAAGCAATAATC		qPCR
Q-CSN1-F	TGCTGGGTTGGCTCACTTAG		qPCR
Q-CSN1-R	AAACTTGCAAGCGCACAGAG		qPCR
Q-KN1-F	GTGCTGGTGCTGGTGAAGTA		qPCR
Q-KN1-R	TCTCTGTCGGTCAACGAAGC		qPCR
T7-F	TAATACGACTCACTATAGGG		insertion amplification
3 AD-R	AGATGGTGCACGATGCACAG		insertion amplification
